# Dunnigan lipodystrophy syndrome: French National Diagnosis and Care Protocol (PNDS; Protocole National de Diagnostic et de Soins)

**DOI:** 10.1186/s13023-022-02308-7

**Published:** 2022-04-19

**Authors:** H. Mosbah, B. Donadille, C. Vatier, S. Janmaat, M. Atlan, C. Badens, P. Barat, S. Béliard, J. Beltrand, R. Ben Yaou, E. Bismuth, F. Boccara, B. Cariou, M. Chaouat, G. Charriot, S. Christin-Maitre, M. De Kerdanet, B. Delemer, E. Disse, N. Dubois, B. Eymard, B. Fève, O. Lascols, P. Mathurin, E. Nobécourt, A. Poujol-Robert, G. Prevost, P. Richard, J. Sellam, I. Tauveron, D. Treboz, B. Vergès, V. Vermot-Desroches, K. Wahbi, I. Jéru, M. C. Vantyghem, C. Vigouroux

**Affiliations:** 1 Endocrinology, Diabetology and Reproductive Endocrinology Department, Assistance Publique-Hôpitaux de Paris, Saint-Antoine University Hospital, National Reference Center for Rare Diseases of Insulin Secretion and Insulin Sensitivity (PRISIS), Paris, France; 2 Sorbonne University, Inserm UMR_S938, Saint-Antoine Research Centre, Institute of Cardiometabolism and Nutrition, Paris, France; 3 Plastic Surgery Department, Assistance Publique-Hôpitaux de Paris, Tenon Hospital, Paris, France; 4 Department of Genetics, Assistance Publique-Hôpitaux de Marseille, Marseille, France; 5 Pediatric Endocrinology Unit, Bordeaux University Hospitals, Bordeaux, France; 6 Nutrition Department, Assistance Publique-Hôpitaux de Marseille, La Conception Hospital, Marseille, France; 7 Paediatric Endocrinology Department, Assistance Publique-Hôpitaux de Paris, Necker Hospital, Paris University, Paris, France; 8 Assistance Publique-Hôpitaux de Paris, Pitié-Salpêtrière Hospital, Myology Institute, Sorbonne University, Paris, France; 9 Paediatric Endocrinology Department, Assistance Publique-Hôpitaux de Paris, Robert Debré Hospital, Paris University, Paris, France; 10 Cardiology Department, Assistance Publique-Hôpitaux de Paris, St Antoine Hospital, Sorbonne University, Paris, France; 11 Endocrinology Department, Nantes University Hospitals, Guillaume et René Laennec Hospital, Nantes University, Nantes, France; 12 Plastic Surgery Department, Assistance Publique-Hôpitaux de Paris, St Louis Hospital, Paris University, Paris, France; 13 French Lipodystrophy Association (AFLIP; Association Française des Lipodystrophies), Pierrevert, France; 14 Sorbonne University, Inserm UMR_S933, Paris, France; 15 Paediatric Endocrinology Department, Rennes University Hospitals, South Hospital, Rennes, France; 16 Endocrinology Department, Reims University Hospitals, Robert Debré Hospital, Reims, France; 17 Endocrinology Department, Lyon University Hospitals, South Lyon Civil Hospital, Lyon University, Pierre Benite, France; 18 Molecular Biology and Genetics Department, Assistance Publique-Hôpitaux de Paris, Saint-Antoine University Hospital, Paris, France; 19 Hepatology Department, Lille 2 University Hospitals, Lille University, Lille, France; 20 Endocrinology Department, La Reunion University Hospitals, Reunion South Hospital, St Pierre de la Reunion, France; 21 Hepatology Department, Assistance Publique-Hôpitaux de Paris, Saint-Antoine Hospital, Sorbonne University, Paris, France; 22 Endocrinology Department, Rouen University Hospitals, Bois-Guillaume Hospital, Rouen, France; 23 Cardiogenetics and Myogenetics Department, Assistance Publique-Hôpitaux de Paris, Pitie Salpêtrière Hospital, Sorbonne University, Paris, France; 24 Rhumatology Department, Assistance Publique-Hôpitaux de Paris, Saint-Antoine Hospital, Sorbonne University, Paris, France; 25 Endocrinology Department, Clermont-Ferrand University Hospital, Clermont Auvergne University, Clermont-Ferrand, France; 26 Endocrinology-Diabetology Department, Dijon University Hospital, François Mitterand Hospital, Bourgogne University, Dijon, France; 27 Cardiology Department, Assistance Publique-Hôpitaux de Paris, Cochin Hospital, Paris University, Paris, France; 28 Endocrinology Department, Lille 2 University Hospitals, Lille University, Lille, France

**Keywords:** Type 2 familial partial lipodystrophy, Dunnigan syndrome, Dunnigan disease, Insulin-resistant diabetes, Diagnosis, Recommendation, Management

## Abstract

Dunnigan syndrome, or Familial Partial Lipodystrophy type 2 (FPLD2; ORPHA 2348), is a rare autosomal dominant disorder due to pathogenic variants of the *LMNA* gene. The objective of the French National Diagnosis and Care Protocol (PNDS; Protocole National de Diagnostic et de Soins), is to provide health professionals with a guide to optimal management and care of patients with FPLD2, based on a critical literature review and multidisciplinary expert consensus. The PNDS, written by members of the French National Reference Center for Rare Diseases of Insulin Secretion and Insulin Sensitivity (PRISIS), is available on the French Health Authority website (in French). Dunnigan syndrome is characterized by a partial atrophy of the subcutaneous adipose tissue and by an insulin resistance syndrome, associated with a risk of metabolic, cardiovascular and muscular complications. Its prevalence, assessed at 1/100.000 in Europe, is probably considerably underestimated. Thorough clinical examination is key to diagnosis. Biochemical testing frequently shows hyperinsulinemia, abnormal glucose tolerance and hypertriglyceridemia. Elevated hepatic transaminases (hepatic steatosis) and creatine phosphokinase, and hyperandrogenism in women, are common. Molecular analysis of the *LMNA* gene confirms diagnosis and allows for family investigations. Regular screening and multidisciplinary monitoring of the associated complications are necessary. Diabetes frequently develops from puberty onwards. Hypertriglyceridemia may lead to acute pancreatitis. Early atherosclerosis and cardiomyopathy should be monitored. In women, polycystic ovary syndrome is common. Overall, the management of patients with Dunnigan syndrome requires the collaboration of several health care providers. The attending physician, in conjunction with the national care network, will ensure that the patient receives optimal care through regular follow-up and screening. The various elements of this PNDS are described to provide such a support.

## Summary for the attending physician

### Introduction

Dunnigan syndrome, or Familial Partial Lipodystrophy type 2 (FPLD2), is a rare autosomal dominant genetic disorder belonging to the large group of laminopathies, diseases related to pathogenic variants of the *LMNA* gene that encodes lamin A/C, proteins in the envelope of the cell nucleus. The most common genetic variant responsible for FPLD2 results in a substitution of the Arg amino acid at position 482. Other pathogenic variants of *LMNA* are responsible for heart disease, myopathy and/or premature aging syndromes. An overlap between these clinical forms is possible. Dunnigan syndrome is characterized by subcutaneous adipose tissue loss from trunk, buttocks and limbs; fat accumulation in neck, face, axillary and pubic regions; muscular hypertrophy and is frequently associated with metabolic and cardiovascular complications Its prevalence, probably largely underestimated, is assessed at 1 case/100,000 inhabitants in Europe [1, 2].

### Diagnosis

*Clinical description* The clinical examination, including a physical examination in underwear, is key for diagnosis. This can be done by a general practitioner. The onset of lipodystrophy (abnormalities in adipose tissue distribution) is usually at puberty and is often more pronounced in women than in men [3, 4]. Therefore, a physician may more easily reach diagnosis in a female index case. This dysmorphic condition is associated with atrophy of subcutaneous adipose tissue of the limbs and trunk (lipoatrophy) with an accumulation of adipose tissue in the face, neck and intra-abdominal region, as well as in axillary and pubic regions. Clinical presentation may suggest Cushing’s syndrome. The subcutaneous lipoatrophy reveals apparent veins and accentuates muscle mass (e.g. hypertrophic calves). At the peri-humeral level, when arms are extended crosswise, it reveals a hatchet deformity of the bicipital mass. The general morphotype is pseudo-athletic with broad shoulders. The BMI is usually normal. *Acanthosis nigricans* is common. Family history of this dysmorphic lipodystrophy contributes to the diagnosis of FPLD, as does the occurrence of cardiac complications in the family (sudden cardiac death, pace-maker-defibrillator) and/or early metabolic complications (diabetes, hypertriglyceridemia).

*Biological* Biochemical testing reveals hypertriglyceridemia, low HDL-cholesterol, hyperinsulinemia, glucose intolerance or diabetes, increased transaminases (hepatic steatosis), CPK can sometimes be elevated (muscle damage) and leptin (adipose tissue hormone) levels are low (leptinemia). In women, hyperandrogenism is common.

*Genetic* Once a familial partial lipodystrophy syndrome is suspected, the patient will be referred to a specialist for a genetic screening of the *LMNA* gene and/or genes involved in others FPLD syndromes. Once a genetic diagnosis has been confirmed, relatives can be offered genetic testing, from about the age of 10, depending on the severity of the familial form.

Patients should receive regular screening and multidisciplinary monitoring; in France, this is done in conjunction with a PRISIS reference/competence center (*PRISIS; Pathologies Rares de l’insulino-Sécrétion et de l’Insulino-Sensibilité. Rare Diseases of Insulin Secretion and Insulin Sensitivity*), particularly regarding metabolic and cardiac complications, which are sometimes present.

### Complications

*Metabolic* Hypertriglyceridemia may appear early (from puberty onwards) and can develop in flare-ups responsible for pancreatitis exacerbated by dietary deviations, estrogens from a contraceptive pill or pregnancy. Impaired glucose tolerance associated with insulin resistance frequently progresses to diabetes in adulthood, but may occur from puberty onwards.

*Cardiovascular* Cardiovascular complications can be major and an ECG, as well as an echocardiography should be included in the work-up towards and in the monitoring after diagnosis. Two types of complications are common:

- Early atherosclerosis: a complete arterial work-up in these patients is recommended from the age of 30 onwards and depending on the variant and family history should include a non-invasive search for myocardial ischemia.
- Dilated cardiomyopathy with conduction disorders, rhythm disorders and/or heart failure may develop in some forms of FPLD2.

Collaboration with a cardiologist, aware of the disease’s cardiac complications, is therefore essential.

*Hepatic* Steatosis is frequent and requires periodic screening to limit complications such as fibrosis and cirrhosis. Following diagnosis, biological and morphological hepatic tests are performed with an annual follow-up, depending on the situation.

*Gyneco-obstetrical* A polycystic ovary syndrome complicating insulin resistance is common. A regular gynecological examination is necessary, as spaniomenorrhea, hirsutism, possible fertility disorders and pregnancies with a metabolic risk are frequent features. Estrogen-progestogens contraception is contraindicated as it increases the risk of hypertriglyceridemia. Contraception should be oriented towards progestogens or an intrauterine device.

*Neuromuscular and rheumatological* Muscular dystrophy, tendon retractions, amyotrophy, muscle weakness, joint or muscle pain are present in some forms of FPLD2.

### Treatment

In France, the social security system completely covers the diagnostic work-up for Dunnigan syndrome (ALD31 procedure). All complications must be investigated and monitored over time. The need for psychological support should be assessed from early on. Metabolic treatment is based first and foremost on diet and increased physical activity. Medical treatments may combine: 1. hypolipidemic drugs (statins, fibrates) with the objectives of a patient at high cardiovascular risk; 2. metformin treatment starting at the glucose intolerance stage; 3. other diabetes treatments if necessary; 4. insulin administered in multi-injections or by subcutaneous pump with sometimes high flow rates or difficulties at the injection site due to the deficit in subcutaneous adipose tissue. 5. In some cases, metreleptin, a leptin analogue, can be prescribed following consensus in a dedicated expert PRISIS multidisciplinary meeting (*RCP; réunion de concertation pluridisciplinaire*), as a daily subcutaneous injection. 6. Finally reconstructive surgery can be proposed in the most disabled cases under the ALD31 social security coverage. Patients should be informed of the existence of the French patients’ association for lipodystrophy patients (AFLIP). Ideally, they participate in therapeutic education programs at reference and competence centers belonging to the rare national network PRISIS. A request for disability recognition by the Regional Centers for Disabled Workers (MDPH, *Maisons Départementales des Personnes Handicapées*) may be useful.

### Conclusion

The disease management of patients diagnosed with Dunnigan syndrome requires the collaboration of several health care providers. The attending physician, in addition to screening and interpreting the disease course, will ensure that the patient receives optimal medical and social care, supported by the PRISIS network. The various elements of this PNDS can be used for support.

## Introduction

Dunnigan syndrome, or Familial Partial Lipodystrophy type 2 (FPLD2), is a rare genetic disorder characterized by:

- partial atrophy of the subcutaneous adipose tissue, affecting the limbs and trunk, in contrast with adiposity of the face and neck, and
- metabolic disorders dominated by insulin resistance and hypertriglyceridemia.

Dunnigan syndrome belongs to the group of laminopathies, which are rare diseases related to pathogenic variants in the *LMNA* gene encoding type A lamins. These proteins, expressed in the nuclei of most differentiated cells, are involved in the regulation of gene expression and cell differentiation. Laminopathic phenotypes are highly variable depending on the pathogenic variant involved and include partial lipodystrophy syndromes, heart disease, myopathies, neuropathies, accelerated aging syndromes. This PNDS will focus on the Familial Partial Lipodystrophy, Dunnigan type and its possible skeletal or cardiac muscle complications.

Dunnigan syndrome is inherited in an autosomal dominant manner and the risk of disease transmission from an affected parent is 50%. The molecular diagnosis is based on the identification of a pathogenic variant of *LMNA*, and is mainly due to a substitution at codon 482 of the gene ((p.R482W/Q or L mutation).

The prevalence of Dunnigan syndrome is not well known. It has been estimated at about 1 case/100,000 population in Europe, but the disease is likely under-diagnosed [1, 2].

Apart from the lipodystrophy morphotype, the metabolic phenotype of the disease, progressively setting in from puberty onwards, is not very specific (insulin resistance, diabetes, hypertriglyceridemia, hepatic steatosis, polycystic ovary syndrome in women), often resulting in a delay in correct diagnosis and management.

The diagnosis of Dunnigan syndrome is covered by the French social security system (ALD31 procedure) and the implementation of multidisciplinary follow-up aims to limit the progression of complications and to detect associated co-morbidities as early as possible. It is therefore important to be able to recognize the particularities of this multi-systemic disease [4].

## Objectives of the national diagnostic and care protocol

The aim of this PNDS is to explain to health care professionals the current optimal diagnostic and therapeutic management and integrated care pathway (ICP) for patients with Dunnigan lipodystrophy syndrome. The goal is to optimize and harmonize treatments and follow-up of this rare disease.

It also identifies pharmaceutical specialties used in an indication not provided for in the marketing authorization, as well as the specialties, products or services necessary for the management of patients but not usually paid for or reimbursed.

This PNDS can be used as a general reference for the general practitioner in cooperation with other medical specialists.

However, the PNDS cannot consider all specific cases, all comorbidities or complications, all therapeutic peculiarities, all hospital care protocols, etc., cannot claim to be exhaustive in regards to treatment, and cannot be a substitute for the individual responsibility of the physician. The protocol does, however, describe a recommended treatment for patients with Dunnigan syndrome. It needs to be periodically updated, based on new and validated data.

This PNDS was drawn up using the "Methodology for the national protocol for the diagnosis and care of rare diseases" published by the French National Health Authority (HAS) in 2012 (technical guide available on the HAS website: http://www.has-sante.fr (in French).

A more detailed document used as a basis for the development of the PNDS, including an analysis of the bibliographic data identified (scientific argument) is available on the HAS website (http://www.has-sante.fr).

## Initial diagnosis and evaluation

### Objectives

- Enable the earliest possible diagnosis of Dunnigan syndrome
- Guide the process of screening for various complications
- Assist in the implementation of therapeutic management in different stages of the disease
- Identify the key educational skills to be acquired by the patient
- Indicate how to organize family screening
- Identify tools for managing psychological, socio-professional and intimate impact of the disease
- Provide contact information for patient organizations
- Present the modalities for periodic monitoring of the disease
- Complete the form for 100% coverage (ALD31 procedure) upon diagnosis

### Professionals involved (and coordination)

Dunnigan syndrome is most often diagnosed by a a general practitioner, endocrinologist or diabetologist in the presence of a suggestive morphotype and metabolic disorders.

Nevertheless, depending on the symptoms reported by patients or families, other specialists may suspect a FPLD diagnosis, which will have to be confirmed by subsequent molecular genetic analysis, with the patient’s consent.

The initial assessment and management of the patient and his/her family require multidisciplinary cooperation involving various specialists (including endocrinologist/diabetologist, cardiologist, neuromyologist, rheumatologist, gynecologist, nephrologist, ophthalmologist, dermatologist, hepato-gastroenterologist, pediatrician, geneticist, orthopedist, plastic surgeon, radiologist, biologist, occupational physician) and other professionals from the paramedical and social fields (pharmacist, nurse, psychologist, dietician, physiotherapist, chiropodist, social worker, others), as needed.

These professionals work together with the general practitioner to provide comprehensive patient care.

### Diagnostic circumstances/diagnostic criteria

#### Clinical diagnostic criteria and initial assessment

The motives for initial consultation may vary. Most often, the disease is evoked in a young woman reporting complaints related to her morphotype, menstrual disorders or hirsutism, sometimes with a painful syndrome (myalgia, pain in the hands in particular) [5]. It is necessary to consider the FPLD diagnosis and search for clinical lipodystrophy in a (young) non-overweight subject, with clinical and biological signs of insulin resistance and a family history, with glucose tolerance disorders or atypical diabetes (without autoantibodies). More rarely and in the case of complex forms of laminopathy, the disease may also reveal itself by cardiac or myopathic signs. The suspicion of for Dunnigan syndrome is clinical. It is based on careful questioning and clinical examination [4, 6].

##### Family context

The examination of family and personal history is essential and should include questioning on:

- not only morphological disorders,
- but also metabolic disorders (diabetes, hypertriglyceridemia, acute pancreatitis),
- early atherosclerosis (ischemic heart disease, peripheral arterial disease, stroke or transient ischemic attack),
- and cardiomyopathy and/or rhythmic and conductive disorders (sudden death in the family, heart failure, pacemaker or defibrillator implantation, or neuromuscular disease),

A family tree summarizing the history of disease and/or complications is useful to determine the familial nature of the disease.

##### Lipodystrophy

Lipodystrophy is generally discovered during clinical examination from the peripubertal period onwards in women; it is generally more discrete in men [4, 7]. Dunnigan syndrome is associated with atrophy of the subcutaneous adipose tissue of the limbs and trunk (lipoatrophy) and an accumulation of adipose tissue in the face and neck, as well as in the axillary and pubic regions. The face may take on a cushingoid appearance with a double chin, filling of the supra-clavicular recesses and sometimes a buffalo hump. Lipoatrophy, which can be present from childhood, accentuates muscle mass and increases the visibility of veins. At the peri-humeral level, it causes a hatchet deformity of the bicipital mass when the arms are extended crosswise. The general morphotype is pseudo-athletic, often with hypertrophic calves, very accentuated buttocks, underdeveloped breasts and a flat stomach (Appendix 1) [8] However, the abdominal muscle wall may be amyotrophic, leading to abdominal protrusion, especially due to an accumulation of intra-abdominal fat (visceral fat) and, frequently, steatotic hepatomegaly. Small subcutaneous lipomas may be present on the abdomen and/or limbs [6].

The body mass index is usually normal. Measuring the thickness of the subcutaneous folds with an adipometer (Harpenden caliper) can help in the diagnosis; in women, values of less than 7 mm at the tricipital level in the upper limb are suggestive of lipoatrophy in the absence of malnutrition.

##### Other dysmorphic and/or examination elements

In women, shoulders are often wider than the pelvis, and legs are often shorter than the length of the trunk would suggest. The extremities, especially the hands, are generally small and wide with short, slightly infiltrated fingers. *Acanthosis nigricans* is common, especially in the axillae and friction areas (belt, nape of the neck), as well as acrochordons (molluscum pendulum), especially in the axillae [9].

##### Neuromuscular and osteoarticular signs

Muscle hypertrophy is ubiquitous in Dunnigan syndrome [10]. It predominates in the limbs and is clinically enhanced by lipoatrophy, particularly in the calves. There may be functional symptoms, such as myalgia, cramps, moderate limb-girdle muscle deficits and tendon retractions (see “Neuromuscular and rheumatological complications” section).

In terms of peripheral nerve damage, nerve compression syndromes are frequent and may be multifocal (carpal tunnel syndrome in the wrist, ulnar tunnel in the elbow, tarsal tunnel, meralgia paresthetica in the thigh). Signs of polyneuropathy may also be associated with diabetes.

Non-specific mechanical rheumatological symptoms (arthralgia, osteoarthritis, tendinopathy) may be observed, without specificity on imaging.

##### Cardiac signs

The heterogeneous and complex cardiac signs associated with Dunnigan syndrome depend on both the severity of metabolic complications and type of *LMNA* pathogenic variant [11, 12].

They can affect prognosis in these patients and result in early cardiologic management [13–15]. The classic cardiovascular risk factors should be monitored. These affect, in addition to the direct consequences of pathological lamin A/C expression also on the cardiac vessels, valves and the heart muscle [14].

The medical history and clinical examination should look for palpitations, including in childhood, malaise with or without loss of consciousness, angina, dyspnea (NYHA classification), intermittent claudication, vascular murmur, abolition of peripheral pulses. Blood pressure should be measured systematically and may be supplemented by outpatient measurements if needed. The use of tobacco should be documented.

The recommended cardiovascular investigations are presented in “Cardiovascular complications” section.

##### Gynecological signs

Polycystic ovary syndrome is common, as is spaniomenorrhea or even amenorrhea, hirsutism, acne, and a variable degree of infertility. Gynecological and obstetric complications will be discussed in “Gynecological complications, fertility/reproductive function” section.

#### Indicative biological elements

The metabolic abnormalities most commonly encountered in Dunnigan syndrome are:

- Hypertriglyceridemia > 1.5 g/L
- Low HDL-cholesterol < 0.5 g/L in women and < 0.4 g/L in men
- Hyperinsulinemia (taking into account the patient’s pubertal stage and BMI), carbohydrate intolerance or diabetes; these abnormalities are sometimes only detected by oral hyperglycemia with two-hour blood glucose and insulin levels, which should be carried out in all cases of suspected Dunnigan syndrome without known diabetes.

The presence of type 1 diabetes auto-antibodies should be excluded. C-peptide is generally detectable or even very high in relationship with hyperinsulinism in FPLD2 patients with diabetes.

An increase in TGP transaminases (ALT), generally higher than TGOs (AST), is common and associated with hepatic steatosis [16]. CPKs may be moderately elevated, but rarely above 2 to 3 times baseline. Leptin is low in relation to BMI. Serum adiponectin is low. Hyperandrogenism of ovarian origin is often diagnosed in women.

#### Imaging

##### Adipose tissue

Dual energy X-ray absorptiometry (DEXA) quantification of total and segmental body fat percentage can be useful to assess the abnormal distribution of adipose tissue [17]. In women:

- a ratio over 1.2 of trunk fat mass to lower limb fat mass,
- a fat mass value of the lower limbs below 25% of total fat mass, are both suggestive of partial lipodystrophy.

MRI, with spectroscopy, if available, can demonstrate accumulation of intra-abdominal fat, > 50% of total abdominal fat mass (normal about 25%).

##### Other organs

Targeted organs imaging is used to explore co-morbidities and complications associated with Dunnigan syndrome. These are detailed in “Assessment of disease complications and comorbidities” section.

Pancreatic imaging (CT or MRI) is prescribed in case of clinical or biological suspicion of acute pancreatitis (epigastric pain, increased serum lipase).

Adrenal imaging (CT or MRI) may be performed in the context of severe or rapidly progressive hyperandrogenism.

### Genetic confirmation of the diagnosis

#### Mode of transmission, gene involved and populations at risk

The Dunnigan syndrome or FPLD2 is referenced in the OMIM and ORPHANET classification under numbers #151,660 and ORPHA2348, respectively. Confirmation of diagnosis of Dunnigan syndrome is based on molecular genetic analysis. Dunnigan syndrome is an autosomal dominant disease caused by pathogenic variants in the *LMNA* gene encoding lamin A/C, a protein of the nuclear envelope.

Although Dunnigan syndrome affects both men and women, the latter generally present more severe clinical forms, both in terms of lipodystrophy and metabolic complications [15]. Some individuals, most often men, may remain asymptomatic or pauci-symptomatic for a long period or do not feel the need to consult a doctor. This should be taken into consideration when inquiring about family history.

#### Nature and interpretation of identified variants

In most forms of Dunnigan syndrome, the genetic diagnosis reveals a heterozygous missense pathogenic variant of the *LMNA* gene. Several dozen pathogenic variants have been described in different exons of the gene, but in more than half of Dunnigan syndrome cases, the variant affects Arginine 482 (p.Arg482Trp and p.Arg482Gln most frequently and p.Arg482Leu less often) [15, 18]. The pathogenicity of the variants is established according to the international recommendations of the ACMG (American College of Medical Genetics and Genomics) [19]. To date, there is no functional test used in routine hospital practice to demonstrate the pathogenicity of a given *LMNA* variant in vitro.

The *LMNA* gene is involved in other monogenic diseases, especially Emery-Dreifuss muscular dystrophy, certain dilated cardiomyopathies, type-2B1 Charcot-Marie-Tooth neuropathy, type-A mandibuloacral dysplasia, restrictive dermopathy, Hutchison-Gilford progeria, as well as in numerous overlapping phenotypes between these different entities [20–22]. Depending on the nature and location of the *LMNA* molecular abnormality, the patient may present a subset of manifestations characteristic of these various syndromes [23]. An online database called UMD-*LMNA*, dedicated to the *LMNA* gene (http://www.umd.be/LMNA/), lists the variants identified in patients, their phenotypes and the associated bibliographic references.

In rare occasions (< 5% according to UMD-*LMNA*), most often due to consanguineous unions, pathogenic variants affecting both alleles of the *LMNA* gene have been identified and are associated with more severe phenotypes of the disease.

#### Diagnostic value of *LMNA* gene analysis

Not all patients with symptoms suggestive of Dunnigan syndrome carry a pathogenic variant in the LMNA gene. Most often, *LMNA* gene analysis is proposed along with a panel of genes involved in lipodystrophy syndromes. In some cases, another genetic cause of lipodystrophy can be identified.

#### Procedures for molecular analysis

The prescription of a molecular analysis of the *LMNA* gene must be carried out in accordance with the rules of good practice in constitutional genetics, such as those validated by the HAS and defining the modalities relating to the prescription, patient information,signed free and informed consent, management of the analyses, communication of the result and information towards relatives (legifrance.gouv.fr). Molecular analysis requires the prior establishment of a family tree.

### Differential diagnosis

Metabolic syndrome, very prevalent in the general population, represents the most common differential diagnosis of Dunnigan syndrome, as disease-specific lipoatrophy is often unrecognized or may be considered clinically as an inconsequential android morphotype, especially in men.

Cushing syndrome is clinically differentiated from FPLD2 by amyotrophy of the limbs and buttocks, truncal and abdominal accumulation of adipose tissue, and facial erythrosis (see PNDS Cushing Syndrome). Acromegaly may also be a differential diagnosis due to the muscular hypertrophy and facial thickening.

Dunnigan syndrome must be differentiated from other lipodystrophy syndromes. The partial nature of the lipoatrophy, the peripubertal appearance of the cushingoid facies and the autosomal dominant transmission of the disease generally differentiate Dunnigan syndrome from congenital generalized lipodystrophies, which are most often diagnosed in early childhood with a consanguineous family history. The differential diagnosis of Dunnigan syndrome with other genetic forms of partial lipodystrophies can sometimes only be revised after the molecular analysis of a dedicated lipodystrophy gene panel. Finally, there are acquired forms of partial lipodystrophy, which may be associated with dysimmune manifestations, in particular childhood panniculitis and/or autoimmune hepatitis [4].

### Announcing the diagnosis and informing the patient

The diagnosis of a lipodystrophy syndrome must be announced to the patient during a consultation with the physician who prescribed the genetic analysis. The molecular genetic report should be explained and given to the patient in person. The patient must be informed of the monitoring and treatment strategy and of the importance of a family investigation. The announcement will take the general context of the patient into account, and the information will be addressed in several stages in conjunction with the attending physician. In addition to medical support, psychological support should be considered whenever necessary. In France, it is recommended for the patient to be referred to one of the centers of the PRISIS reference network, (see Appendix 2). The patient must be informed of the existence of the French Lipodystrophy Association (AFLIP, Association Française des Lipodystrophies) and, if necessary, associations of patients with diabetes (AJD, Aide aux Jeunes Diabétiques; FFD, Fédération Française des Diabétiques) (Appendix 2) and/or of other International Lipodystrophy and/or Diabetes Associations. The attending physician will complete the form for 100% coverage (ALD31, see “Therapeutic care” section).

### Genetic counselling and genetic screening

#### Genetic counselling

The doctor who delivers and explains the results of the genetic analysis to the patient should also provide advice regarding the best suited health professional to the patients care. Additional genetic counselling may be required, especially to assist in screening relatives, for family members in pre-symptomatic screening of children or in case of family planning and pregnancy.

#### Screening of relatives

As the diagnosis of Dunnigan syndrome may lead to preventive and/or curative measures, the physician prescribing the genetic analysis must inform, in coordination with the patient, her/his adult relatives in accordance with the autosomal dominant transmission of the disease and the modalities provided by the French legislation (Article L1131-1–2 of the French Public Health Code). If the patient does not wish to inform her/his family members, the doctor will offer to send an information letter. This letter will mention the existence of a family disease that requires a genetic consultation without disclosing the name of the person who underwent the initial examination, the name of the genetic disease or its associated risks.

#### Pre-symptomatic diagnosis

Even though the disease course can be more or less severe, particularly depending on gender and lifestyle, Dunnigan syndrome has almost complete penetrance, i.e. a subject has a near 100% risk of developing the symptoms if she/he carries the pathogenic family genetic variant. Furthermore, an anticipation phenomenon has been reported in Dunnigan syndrome describing an earlier and more severe onset of metabolic complications over several generations, that could be in part favored by environmental factors [23]. Lastly, cardio-metabolic complications, which could develop later, require preventive screening, monitoring and treatment and should be introduced as early as possible (see “Therapeutic care” section).

Based on family context and clinical phenotype, the physician may propose a pre-symptomatic genetic screening in children. Genetic analysis can be proposed from 10 years onwards [13, 23], in order to manage the cardiometabolic follow-up accordingly. In accordance with the French legislation, the genetic analysis should be prescribed by a doctor belonging to a multidisciplinary team registered at the French Biomedicine Agency. In the absence of genetic screening, regular clinical and biological monitoring of asymptomatic relatives should be offered at the metabolic level (blood sugar, lipids), particularly before contraception is prescribed. Similarly, a familial cardiac involvement should lead to a systematic cardiac monitoring (ECG, cardiac ultrasound and Holter recording) from the age of 10, as recommended for the management of relatives of patients with dilated cardiomyopathy [13, 23].

#### Prenatal diagnosis

If requested by the parents, in particular in the presence of severe cardiomyopathy associated with Dunnigan syndrome, the indication of a prenatal diagnosis may be discussed on a case-by-case basis. This requires that one of the parents of the unborn child carries a genetic variant which has been clearly identified as responsible for the disease. In this case, a Multidisciplinary Center for Prenatal Diagnostics (CPDPN, Centre pluridisciplinaire de diagnostic prénatal) should validate or not the indication, in conjunction with a Department of Medically Assisted Reproduction.

### Assessment of disease complications and comorbidities

The recommended initial assessment is summarized in Appendix 3 and follow-up in Appendix 4.

#### Metabolic complications: diabetes, hypertriglyceridemia, risk of acute pancreatitis

Metabolic disorders (insulin resistance, impaired glucose tolerance, hypertriglyceridemia [24] may reveal the nature of the disease and should be systematically investigated (see “Indicative biological elements” section). Hypertriglyceridemia can develop from as early as the prepubertal period, especially in girls [3] Hypertriglyceridemia flare-ups can be complicated by potentially recurrent acute pancreatitis, which is exacerbated by dietary deviations, estrogen-progestin contraception or pregnancy. Impaired glucose tolerance associated with insulin resistance can progress to diabetes, most often in puberty or adulthood.

The diagnosis of diabetes requires investigating microangiopathic complications of the disease based on current recommendations, especially with an annual fundus examination, microalbuminuria and creatinine measurements. Screening for cardiovascular complications is detailed in “Cardiovascular complications” section.

#### Hepatic complications

The prevalence of liver damage is high: the absence of adipose tissue leads to an ectopic lipid storage, and the liver is a prime target. Liver steatosis is present in more than 80% of patients with Dunnigan syndrome [16]. This can be complicated by non-alcoholic steatohepatitis (NASH), sometimes associated with fibrosis that can progress to cirrhosis [25]. Diagnosis should be made early to prevent complications of liver fibrosis [4, 26, 27].

Clinical examination looks for hepatomegaly suggestive of hepatic steatosis. The presence of clinical signs of cirrhosis prompts a specific management by the hepatologist.

Initial and annual monitoring of transaminases, alkaline phosphatase, GGT, total bilirubin, albumin, platelet and hemostatic functions are recommended. Even though non-invasive biological markers of hepatic fibrosis (Fib4 score, Aspartate amino transferase-to-Platelet Ratio index (APRI) or morphological markers (elastometry) have not been studied specifically in FPLD2 [28], they are useful as in the general population. The Fib4 score [Age (years) x AST (IU/L)/Platelets(10^9^/L) × √ [ALT (IU/L)] will be measured at the time of diagnosis, then annually.

Ultrasound morphology of the liver is indicated at the time of diagnosis and should be repeated in case of clinico-biological progression in order to detect hepatomegaly, hepatic steatosis (hyperechogenic liver on ultrasound indicates steatosis > 30%), and/or signs of cirrhosis (afef.asso.fr).

In case of hepatomegaly, hepatic biological anomaly, diagnosis of steatosis and/or suspicion of cirrhosis, alcohol consumption should be prohibited. The etiological work-up will be completed by HCV and HBV serologies, measurement of ceruloplasmin, ferritin, anti-smooth muscle, anti-nucleus, anti-LKM, anti-mitochondria, and anti-transglutaminase IgA autoantibodies, and TSH, and elastometry [29].

In case of Fib4 score > 4, F2 classification at elastometry [28], cytolysis with AST or ALT > 2N, and/or signs of cirrhosis and/or portal hypertension on ultrasound, the patient will be referred to an hepatologist, if possible from a rare liver disease network, in order to evaluate the indication of a liver biopsy (afef.asso.fr) for diagnostic and prognostic purposes, according to current recommendations (HAS-Doctor's Guide to Cirrhosis).

#### Cardiovascular complications

Patients with the typical FPLD2 phenotype associated with *LMNA* p.Arg482 variants are at risk of early atherosclerosis, which can develop even in the absence of diabetes, sometimes before the age of 45 [13].

Patients with complex phenotypes combining lipodystrophy syndrome and skeletal striated muscle and/or cardiac laminopathy, most often related to other pathogenic *LMNA* variants, are at risk for dilated cardiomyopathy, heart failure, conduction disorders, supraventricular and ventricular rhythm disorders and sudden death [11, 12, 30]. The predictive factors for malignant arrhythmias/sudden death are: male gender, non-missense *LMNA* pathogenic variant, atrioventricular block, non-sustained ventricular tachycardia and left ventricular systolic dysfunction [31].

As soon as the diagnosis of Dunnigan syndrome is suspected, a resting electrocardiogram (12-lead ECG) should be performed to search for signs of ischemia, sequelae of infarction and rhythm or conduction disorders (sinus dysfunction, atrioventricular block, bundle branch block, supraventricular or ventricular arrhythmia). A transthoracic echocardiography should be performed to measure the size of the heart chambers, left and right ventricular function, global and segmental kinetics, and to serve as a reference for follow-up. Screening for coronary ischemia by a functional test (exercise or pharmacological stress test) or by a coronary CT-scan should be considered from the age of 30, especially in presence of familial cardiac disease and/or associated cardiovascular risk factors. It should be performed without delay in case of abnormalities at clinical examination or on resting ECG. Vascular doppler ultrasound of supra-aortic trunks, aorta and lower limbs can also be proposed [32].

An ambulatory 24/48 h ECG may be added to first-line cardiovascular investigations, based on clinical signs and examination, resting ECG and family history. The purpose of this examination is to detect paroxysmal rhythm or conduction disorders. It should be performed systematically if the lipodystrophy phenotype is associated with neuromuscular or progeroid elements, or if the causal *LMNA* variant is not a substitution at codon 482 [11]. The purpose of this examination is to look for paroxysmal rhythm or conduction disorders [31, 33]. A cardiac MRI can provide prognostic information by detecting a decrease, even minimal, in the left ventricular ejection fraction or the presence of late gadolinium enhancement associated with myocardial fibrosis (which may affect especially the interventricular septum). This sign could be an early marker of the risk of conduction disorders and ventricular arrhythmias, which usually precedes structural myocardial damage in laminopathies [31]. Regarding biomarkers, CPK measurement is useful to detect potential muscle involvement. NT pro-BNP may be elevated in cases of left ventricular dysfunction or dilatation and is associated with the occurrence of ventricular arrhythmias.

A decision tree for cardiovascular investigations is provided in Appendix 5.

Partial lipodystrophy is also a risk factor for sleep apnea syndrome, which should be systematically investigated clinically (daytime sleepiness, snoring, asthenia, non-restorative sleep, concentration difficulties) and, at the slightest diagnostic doubt, by polysomnographic sleep recording during initial assessment and follow-up [34, 35].

#### Gynecological complications, fertility/reproductive function

Patients with FPLD are frequently described having menstrual cycle abnormalities. However, these studies are based on a small series. More than half of patients with Dunnigan syndrome are likely to have polycystic ovary syndrome (PCOS) (International recommendations, 2018).

Oligo-spaniomenorrhea or amenorrhea, as well as hyperandrogenism, should be investigated in adolescence [36]. Compared to the majority of patients from the general population with PCOS, patients with FPLD generally have a lower body mass index (BMI) [4, 37]. Depending on the clinical data, measurements of FSH, LH, estradiol, total testosterone and Sex-Hormone Binding Globulin (SHBG) may be proposed. Due to FPLD-related insulin-resistance, SHBG is often low, which may lead to underestimation of total testosterone. It is therefore useful to calculate the bioavailable testosterone. The anti-Müllerian Hormone (AMH) level is an indicator of ovarian reserve: it is increased in the case of PCOS and decreased in the case of decreased ovarian reserve.

A pelvic ultrasound (transvaginal when possible), can reveal the presence of ovaries of increased volume and/or with multiple follicles. In addition, these patients with FPLD have a higher risk than the general population of developing ovarian cysts.

There is very little data on the fertility of patients with Dunnigan syndrome. In a series of 14 women with FPLD2, carrying the *LMNA* p.Arg482 variant, infertility was reported in 28% of cases [38].

Infertility in men with Dunnigan syndrome has not been documented in the literature.

Pregnancy in FPLD2 patients may be complicated by miscarriage, gestational diabetes, hypertension and pre-eclampsia, fetal death in utero and prematurity. In addition, the risks of hypertriglyceridemia and acute pancreatitis are amplified during pregnancy [4, 38].

#### Neuromuscular and rheumatological complications

In addition to myalgia and muscle cramps, frequently reported in patients with Dunnigan syndrome, peripheral muscle degeneration or diabetes-associated peripheral nerve damage can be observed.

Dunnigan syndrome can be associated with muscular dystrophy, particularly of the limbs, with retraction of Achilles tendons or elbows, difficulty for standing on heels and the tendency to walk on tiptoes. Amyotrophy leading to a major deficit in muscle strength can be seen in some patients.

Muscle strength deficits may come to light by difficulties in raising the arms when removing clothing, lifting the shoulder blades, waddling when walking and/or with difficulty in rising from a squat or climbing stairs.

Advanced muscle damage may be complicated by a decline in respiratory function due to respiratory muscle damage, which should be assessed by respiratory function testing.

CPKs are often moderately elevated. Muscle MRI (shoulder girdle, calves, quadriceps) may show amyotrophy or fatty degeneration of certain muscle bundles, particularly in the thighs, while the calf muscles are hypertrophied with an almost total disappearance of subcutaneous fatty tissue. An electromyogram confirms myopathic damage [39], sometimes revealing myogenic disturbances. A muscle biopsy is discussed in a specialist consultation.

In case of nerve compression syndromes or diabetic neuropathy, the electromyogram may show a decrease in conduction velocities and/or a lengthening of distal latencies, in addition to other clinical signs: paresthesia, pain, peripheral sensitivity disorders, abolition of osteotendinous reflexes [40]. Signs of dysautonomia (orthostatic hypotension, lack of variation in heart rate on exercise, delayed gastric emptying, bowel problems, bladder neuropathy, erectile dysfunction in men) may be observed, when diabetes is long-standing and poorly managed.

Concerning rheumatology, the imaging workup is clinically oriented and targets symptomatic areas [41].

#### Particularities in the peripubertal period

Faciocervical lipohypertrophy and peripheral lipoatrophy most often develop from the peripubertal period onwards and are accompanied by metabolic complications. Puberty is a high risk period for diabetes decompensation. Close metabolic and hepatic monitoring is necessary, in order to introduce preventive or therapeutic measures, including the contraindication of estro-progestative contraception. We recommend an early psychological follow-up, as body image can be a source of distress [42]. The onset of signs during the pubertal period and the need for specific contraception may justify a presymptomatic genetic diagnosis from the age of 10; depending on the family context (see “Pre-symptomatic diagnosis” section). In its absence, general information on preventive lifestyle measures and metabolic monitoring will be given.

## Therapeutic care

### Objectives

Dunnigan syndrome is a chronic, progressive and systemic disease. It usually requires more than six months of treatment, which can be particularly expensive. As a result, 100% ALD31 coverage by the French health social security is required. The objectives of care include:

- Screen for and treat metabolic abnormalities early
- Organize multi-disciplinary management of the disease based on co-morbidities and complications
- Monitor treatment compliance and side effects
- Integrate therapeutic education of the patient and his/her relatives
- Include the patient and his/her family in the decision-making process to enhance trust and adherence to the treatment strategy.
- Discuss participation in a therapeutic trial, if applicable.
- Explain molecular test results and arrange for family screening at the time of diagnosis and during follow-up.
- Evaluate quality of life, self-esteem, social relationships, psychological repercussions, school or socio-professional consequences. Improve quality of life in the pediatric and adult ages, as well as during pregnancy.
- Propose psychological follow-up if deemed necessary.
- Propose aesthetic treatment if needed.
- Refer patients and family to self-help groups and/or patient associations.
- Organize follow-up with the primary care physician.
- Contact with school or occupational physician, if necessary.
- Assess, together with the patient, potential need for disability recognition by the Regional Centers for Disabled Workers (http://www.firendo.fr/prise-en-charge-du-patient/vivre-avec-une-maladie-rare/).

### Professionals involved (and coordination arrangements)

Treatment is multidisciplinary and coordinated by a general practitioner, specialist physicians and professionals from the paramedical and social field (see “Professionals involved (and coordination)” section). The referring specialist is usually an endocrinologist/diabetologist. Nurses and dieticians, especially those involved in therapeutic education and/or compliance monitoring, have an important role to play in implementing treatment and follow-up.

The indication and follow-up of treatment with metreleptin should be discussed according to the recommendations of the HAS in coordination with the PRISIS Reference Network. Collaboration should be established with the pharmacist responsible for dispensing the treatment (see Appendix 6 and “Metreleptin” section).

### Therapeutic care

#### Diet and lifestyle

Treatment is first and foremost based on lifestyle and dietary measures, which should be implemented as soon as a FPLD is suspected, before complications may appear and, if possible, in collaboration with a dietician. In lipodystrophy, the reduced capacity of adipose tissue to store excess energy plays an important role in the development of metabolic alterations. It is therefore necessary to avoid any situation in which food energy intake exceeds energy expenditure.

The recommended nutritional intake and nutrient repartition is as follows (ANSES PNNS 2016 and ANSES PNNS 2020 for children between the ages of 4 and 17):

- 40 to 55% carbohydrates with a preference for complex carbohydrates with a low glycemic index (< 55), such as legumes, wheat, quinoa, rice and whole-wheat pasta.
- 35 to 40% lipids with a preference for mono and polyunsaturated fatty acids, including omega 3 (walnut, rapeseed, soya, fatty fish oils) and limiting saturated fatty acids (cold meats, cheese, butter, sour cream).
- 10 to 20% of protein.
- Vegetables are indispensable for their contribution in fibers.
- Simple carbohydrates from sweet products should be limited as much as possible (5% of the serving).

In addition:

- Limitation/avoidance of alcoholic and/or sugary drinks is required to limit risks of hypertriglyceridemia and hepatic steatosis.
- Smoking is strongly discouraged due to the high cardiovascular risk.

These recommendations can be adapted and individualized according to the patient’s physiological context (growth, pregnancy and breastfeeding), his/her BMI, diabetes status and/or hypertriglyceridemia and his/her adherence to the lifestyle-dietary rules.

In case of severe hypertriglyceridemia, a strict diet with no high glycemic index carbohydrates, no alcohol, low fructose, limited unrefined carbohydrates and a < 20% fat restriction is recommended. Caloric restriction can improve metabolic abnormalities.

In malnourished children or adults, powdered preparations, oils and margarines based on medium-chain triglycerides can be used to meet energy needs [43].

Particular attention must be paid to eating disorders (bulimia) which are favored by the dysfunction of the adipose tissue together with leptin deficiency and reinforced by the psychological impact of lipodystrophy.

With regard to physical activity, a cardiovascular examination should be carried out in all subjects with lipodystrophy before starting a physical activity program (see “Cardiovascular complications” section). Patients with lipodystrophy should be encouraged to engage in regular physical activity, at least 3.5 h per week (1 h per day for children; ANSES PNNS 2020), compatible with potential complications, especially cardiovascular, as well as muscular fatigability. Physical activity should be combined with stretching exercises. The type of physical activity will be adapted to the functional capacities of patients (pain, muscle fatigability).

#### Psychological care

Quality of life, self-esteem, social relationships, and school or socio-professional consequences of the FPLD should be assessed [42]. Depending on the situation and how the disease is perceived, psychological care may be necessary, particularly during diagnosis, life events (adolescence, transition from pediatrics to adult medicine, pregnancy) and depending on the acceptance of the disease (perception of self-image, complications). Referring to family and patient organizations can be helpful. A psychological assessment will be integrated into the therapeutic education programs.

#### Drug management of insulin-resistance, hyperglycemia and liver impairment

##### Management of insulin-resistance and hyperglycemia

Metformin treatment can be introduced in case of carbohydrate metabolism abnormality (fasting hyperglycemia > 1 g/l or carbohydrate intolerance with glucose under OGTT > 1.40 g/L) (endorsed off-label prescription).

Treatment of diabetes in the context of lipodystrophies is most often based on multimedication. Metformin and thiazolidinediones (the latter not available in France) are the molecules most commonly used to decrease insulin resistance. However, published efficacy data remain limited. Insulin treatment is frequently used, usually in high doses and/or concentrated formulations (U-200, U-500 through an authorization for temporary use (ATU)), including subcutaneous pumps, monitored by a specialized team. Abnormalities in insulin diffusion with the risk of hypoglycemia could theoretically be observed when Glargine or Degludec insulin is administered in lipoatrophic areas. Indeed, the prolonged duration of these insulin analogues is based on the presence of subcutaneous fat tissue.

There is still very limited published data on other glucose-lowering molecules used in adults, such as GLP-1 analogues, DPP4 or SGLT2 inhibitors. In children, these molecules may also be used under supervision, despite the absence of a marketing authorization.

GLP-1 analogues have been shown to have a beneficial effect on glycemic control in some patients with FPLD2. A reduction in insulin requirements and a favorable impact on hunger and hyperphagia have been reported [44]. In FPLD subjects with normal weight, particular attention should be paid to weight loss [45]. In the absence of significant cohorts of patients with Dunnigan syndrome treated with GLP-1 analogues, the risk of acute pancreatitis, which is primarily dependent on pre-existing hypertriglyceridemia, should be assessed. Nevertheless, a recent meta-analysis does not show an increased risk of acute pancreatitis in type 2 diabetics on GLP1 analogues [46]. Treatment with SGLT2 inhibitors could be promising for their metabolic effects, but also for their impact on cardiovascular, renal and hepatic complications [47].

##### Management of liver damage

Lifestyle and dietary rules are prescribed as a first line treatment. Deoxycholic acid has not been shown to be specifically effective in treating hepatic steatosis [48]. The most significant benefits in patients with FPLD were obtained with metreleptin (see “Metreleptin” section).

#### Drug management of dyslipidemia

Dunnigan syndrome is frequently associated with hypertriglyceridemia, which can be severe and lead to acute pancreatitis, as well as a decrease in HDL-cholesterol. Less frequently, an increase in LDL-cholesterol is noted.

Because of the increased cardiovascular risk in Dunnigan syndrome (high cardiovascular risk category), a target of LDL-cholesterol < 1.00 g/l (2.58 mmol/l) and non-HDL-cholesterol < 1.30 g/l (3.35 mmol/l) is recommended in primary prevention. If Dunnigan syndrome is associated with diabetes or another cardiovascular risk factor, the recommended LDL-cholesterol target is < 0.70 g/L (1.8 mmol/L). In secondary prevention (established cardiovascular disease), the recommended LDL-cholesterol target is < 0.55 g/L (1.4 mmol/L) [49].

Thus, in accordance with the European consensus [49], in association with lifestyle and dietary measures, prescription of a statin as a first-line treatment will often be necessary. The dose and choice of the molecule will be adapted to reach the recommended LDL-cholesterol target. In case of intolerance to statins, ezetimibe can be proposed.

Fibrates should be initiated, as soon as plasma triglycerides exceed 5 g/l (5.65 mmol/l) [4]. If the response is insufficient, omega-3 fatty acid supplementation may be added. Because of the high cardiovascular risk in patients with Dunnigan syndrome, fibrate therapy may also be considered in patients with high triglycerides (> 2.00 g/l or 2.26 mmol/l) and low HDL-cholesterol (< 0.50 g/l or 1.30 mmol/l) in women and < 0.40 g/l (1.00 mmol/l) in men) after having reached the LDL-cholesterol target, in accordance with the European consensus [49]. In this case, it is strongly recommended to use fenofibrate, which has been proven safe in combination with statins. Gemfibrozil should not be used in combination with statins.

Because of potential muscular symptoms (pain, fatigue or confirmed muscular dystrophy) in Dunnigan syndrome, it is recommended to measure CPKs and check liver function before introducing a lipid-lowering treatment. Lipid-lowering therapy should not be initiated if CPK levels are > 5 N and transaminases > 3 N. Routine clinical monitoring of muscle symptomatology during treatment is recommended.

Lipid and liver tests should be performed 2 to 3 months after any initiation of lipid-lowering treatment (statins, fibrates), as well as CPK tests in case of muscle complaints, then once or twice a year during the subsequent follow-up.

#### Metreleptin

Metreleptin is a leptin analogue. It is the only specific treatment for lipodystrophy syndromes to date [47, 50, 51]. It aims to improve the metabolic complications associated with lipodystrophy, which are partly related to adipocyte hormone deficiency, but it does not restore atrophic adipose tissue. It has not been the subject of controlled studies in these rare diseases [52, 53].

In FPLD, metreleptin efficacy on HbA1c, triglyceridemia and liver parameters [54] was mainly observed in patients with complications not controlled by standard treatments, together with low leptinemia in relation to BMI [55]. Metreleptin improves hyperphagia and causes weight loss in leptin-deficient patients, which may also have a favorable effect on the cervico-facial accumulation of adipose tissue in Dunnigan syndrome.

In patients with a confirmed partial lipodystrophy syndrome, metreleptin has obtained marketing authorization in Europe (ema.europa.eu) and in France (ansm.sante.fr), in adjunction to diet, as a replacement therapy to treat complications associated with leptin deficiency:

-in adults and children from 12 years and older,

-in case of metabolic complications insufficiently controlled by standard treatment.

In France, initiation and renewal of metreleptin prescription is restricted to hospital practitioners specialists in pediatrics or endocrinology, diabetes and nutrition.

The French Health Authority (HAS) recommends that leptin indication should be validated, and regularly re-evaluated, during expert multidisciplinary meetings of the PRISIS network. Initial blood leptin level should be documented.

Metreleptin therapy is a daily injectable treatment that is restrictive and expensive. Product characteristics, mode of administration and side effects are summarized in Appendix 6.

#### Cardiovascular management

The primary prevention of atherosclerosis is similar to the first-line treatment of cardiovascular risk factors frequently associated with FPLD. Metabolic complications should be managed according to current recommendations, emphasizing the importance of dietary measures and physical activity. Physical exercise should be encouraged in the absence of cardiac contraindications, starting in adolescence. The management of dyslipidemia and diabetes is discussed in “Drug management of insulin-resistance, hyperglycemia and liver impairment” and “Drug management of dyslipidemia” sections. Arterial hypertension is treated following general population recommendations, but attention should be paid to bradycardia-inducing beta-blockers and certain calcium antagonists, due to the possible existence of a high-grade conduction disorder.

In secondary prevention, the strategy of coronary revascularization is entrusted to expert cardiologists. Management of cardiomyopathy and/or rhythm and conduction disorders relies on cardiologists who are familiar with the management of laminopathy. Indeed, compared to other etiologies of cardiomyopathy, rhythm and/or conduction disorders may come early and precede structural damage to the myocardium. Therefore, an implantable cardioverter defibrillator (ICD) for primary prevention is usually early indicated in cases of *LMNA*-related cardiomyopathy. A risk stratification algorithm for sudden death and malignant arrhythmia has been proposed for primary prevention to optimize the indications for ICD placement (lmna-risk-vta.fr).

After initial assessment, the follow-up by the referring cardiologist is adapted to the patient’s personal and family history and genotype. It should include, at least annually, a clinical examination, blood pressure measurement and 12-lead ECG. In case of confirmed cardiomyopathy and/or rhythm/conduction disorders, the follow-up frequency of transthoracic cardiac echography and Holter ECG 24/48 h will be evaluated in collaboration with the cardiologist.

#### Gynecological care

##### Cycle abnormalities, contraception and fertility

In adolescent and adult women with FPLD2, the management of PCOS is consistent with recent recommendations: lifestyle modifications with weight loss if overweight or obese, and increased physical activity. Metformin, an insulin-sensitizing treatment, can improve the regularity of menstrual cycles and possibly hyperandrogenism.

Anti-androgen treatments should be offered to patients with disabling hirsutism and/or acne. In these patients, the first-line treatment is not the combined estrogen-progestin birth control pill. Indeed, due to frequent hypertriglyceridemia, oral estrogens are contraindicated because of their primary hepatic passage. Depending on the severity of the hyperandrogenism, anti-gonadotropic progestin therapy combined with transdermal estrogen may be prescribed. If cyproterone acetate is used, oral and written information on the risk of meningioma should be provided to the patient and signed by the physician and the patient (ansm.sante.fr). A brain MRI should be done before initiating treatment. The prescription of spironolactone (off-label) can be considered but should be systematically associated with contraception. If hirsutism is pronounced, laser hair removal (electrolysis or photo-epilation) is recommended and should be covered by ALD31 in France. A request for prior agreement will be made to the doctor advising Social Security.

Regarding contraception, FPLD2 patients are at high cardiovascular risk (diabetes, hypertension, dyslipidemia, early atherosclerotic events). Progestin or non-hormonal contraception is recommended.

In FPLD2 patients with anovulatory infertility associated with PCOS, metformin treatment can be offered, but clomiphene citrate treatment is more effective in inducing follicular recruitment and promoting fertility. The benefit of metreleptin treatment on fertility in FPLD2 patients has not been evaluated.

##### Pregnancy

Patients with FPLD2 should be offered a preconception consultation with a medical team familiar with lipodystrophy and diabetes management during pregnancy. During pregnancy, they should be followed jointly in a center specializing in the management of lipodystrophy and preferably in a level 2 or 3 maternity hospital.

During preconception, particular attention should be paid to treatments with potential teratogenicity (statins, fibrates, anti-hypertensive drugs, which should be discontinued). In case of pre-existing diabetes, the pre-conceptional glycated hemoglobin (HbA1c) should be below 6.5%, as in type 2 diabetes. Triglyceride levels should also be measured to establish a baseline before pregnancy. Metformin treatment should be stopped as soon as a positive pregnancy diagnosis is made.

For patients with metreleptin treatment, whether or not to continue this treatment during pregnancy should be discussed, bearing in mind that:

- The teratogenicity of metreleptin is not known
- Discontinuation of the treatment carries a risk related to extreme insulin resistance during pregnancy with foreseeable difficulties in managing diabetes and hypertriglyceridemia, increasing the risk of acute pancreatitis

During pregnancy, in patients without diabetes, fasting blood glucose should be measured as soon as possible. Diabetes may require very large doses of insulin, especially from the 2nd trimester onwards. It is sometimes necessary to resume metformin during the 2nd trimester when insulin treatment is not sufficient (since teratogenicity data are reassuring). Regular monitoring of fetal ultrasound is essential, allowing fetal macrosomia detection. Blood pressure should be monitored at least once a month in order to diagnose potential gestational hypertension or pre-eclampsia. We recommend measuring triglyceride levels every month if they were above 5 g/L before pregnancy onset. A triglyceride concentration above 10 g/L confers a risk of acute pancreatitis. Adapted dietary management is required, avoiding a reduction of lipid intake below the recommendations for pregnant women. For triglyceride values above 10 g/L, treatment with omega-3 fatty acids may be proposed. In case of persistently high triglyceride levels, the advice of an expert center should be sought [49].

The introduction or resumption of metreleptin during pregnancy, if diabetes or hypertriglyceridemia is difficult to control, may be discussed during a multidisciplinary meeting with the expert center.

#### Neuromuscular management

The management of muscular symptoms such as myalgia and muscle cramps is based on analgesic treatment, according to the intensity of the pain, its location, context, associated factors, the patient’s age and co-morbidities, as well as decontracting treatments, analgesic massages and metabolic control.

When Dunnigan syndrome is associated with muscle degeneration, no pathophysiological treatment is available to improve amyotrophy and muscle strength degradation. Current treatment options, to be implemented in coordination with a multidisciplinary neuromuscular consultation, include [56]:

- physical therapy with stretching, massage and gentle muscle strengthening to prevent and stabilize tendon retractions and muscle decline.
- mechanical aids (cane, orthopedic support, walker and wheelchair) as needed to keep walking as long as possible.
- surgery for tendon retractions (especially Achilles) and severe scoliosis, after respiratory capacity evaluation and respiratory physiotherapy if necessary, coughing assistance and non-invasive ventilation.

In addition, influenza and pneumococcal vaccinations should be up to date in patients with a respiratory disease. The use of depolarizing muscle relaxants (succinylcholine) and volatile anesthetics (halothane, isoflurane) should be avoided during surgical procedures in muscle laminopathies (theoretical risk of malignant hyperthermia).

The analgesic treatment for neuropathic pain may include antiepileptic drugs (gabapentin, pregabalin), some antidepressants (clomipramine, imipramine, amitriptyline, duloxetine), and topical medications for localized pain (lidocaine plasters). In case of ductal syndrome, a surgical indication (endoscopy) may be discussed.

#### Plastic surgery

Dunnigan syndrome combines lipoatrophy of the limbs and trunk subcutaneous adipose tissue, with a fat accumulation in the face, neck, and upper trunk. The absence of adipose tissue or its redistribution leads to changes in physical appearance that can be stigmatizing [42]. In women, for example, the reduction or absence of breast tissue induced by lipodystrophy, the muscular hypertrophy of the limbs as well as the cushingoid aspect of the face can have a strong psychological impact, as these morphotype particularities are often experienced as an attack on feminity [57–59]. Conventional drug therapies have no significant effect on the redistribution of adipose tissue. Although little objective data are available, metreleptin treatment may decrease facial fat volume, probably in relation to the loss of fat mass under treatment [60].

In case of significant psychological repercussions in adults, a consultation with a plastic surgeon who is familiar with lipodystrophy may be proposed to the patient. Reconstructive surgery, which can act on the hypertrophic and atrophic components of lipodystrophy, can feminize the body, which is often sought by patients. Areas of fatty hypertrophy, often located on the face, at the roots of the limbs and in the pubic region, are treated by liposuction or surgical resection (dermo-lipectomy). Atrophic areas, mainly in the breasts and limbs, may be treated with autologous fat injections, as performed in patients with HIV-related lipodystrophy ([61], and unpublished personal data in Dunnigan syndrome). The fat may be then transferred from areas of hypertrophy to areas of atrophy. Several surgical procedures are often needed to reach a satisfactory result. Breast implants are also an option for breast augmentation. Reconstructive surgery has no known impact on the metabolic complications of the disease. Diabetes and cardiovascular risk factors should be controlled before surgery, including the interruption of any smoking, so as not to compromise the healing process. Surgical risks must be clearly explained (hematomas, hypoesthesia), as well as the risk of long-term recurrence. In France, the request for coverage by Social Security must be submitted to the Medical Officer for prior agreement under the ALD31 procedure.

### Therapeutic education

Therapeutic education designates a set of activities (awareness, information, learning, psychological and social support) designed to help patients (and his/her family) improve their quality of life, to understand the implications of their diseases and its treatments, to participate in care, take charge of his/her state of health and to promote, as far as possible, the maintenance of daily activities. It takes into account the whole person by evaluating the patient’s personal plans, experience with Dunnigan lipodystrophy and knowledge of the disease. Therapeutic education should ensure that the patient and his/her family do understand information related to the disease and its management.

This approach requires to provide information to patients and their families regarding:

- The disease, its manifestations and warning signs that should lead to a consultation.
- How the disease is transmitted, the importance of family screening and the possibility of genetic counselling.
- Methods of delivering and administering available or potential treatments and their possible adverse effects.
- Follow-up procedures and the course of symptomatic management based on the patient’s needs, emphasizing the importance of regular monitoring assessments.
- Daily life (professional, family, social, travels).
- Financial aid and legal assertion (ALD31, psychological support).
- Existence of institutional websites, such as Orphanet and patient associations.

Patients will acquire various self-care skills during an individualized educational program. This information will be included in subsequent consultations.

A medical Individualized Welcome Project (PAI, *Projet d’Accueil Individualisé*) or Personalized Schooling Project (PPS, *Projet Personnalisé de Scolarisation*) is drawn up with the child’s care structure or the school doctor if necessary.

If the intensity of care requires a rehabilitation program that is not covered by social security, a request for disability recognized by the MDPH can.

be made (ex: psychomotricity). The same is true if the illness causes social or financial consequences for the family (family caretakers, interruption of professional career, continuous glucose monitoring).

### Patient associations

All healthcare professionals and patients should be informed of the existence of patient associations for lipodystrophic patients: the French Lipodystrophy Association (AFLIP; *Association Française des Lipodystrophies*) (https://www.facebook.com/AFLIP02*)*, as well as information regarding international lipodystrophy patient associations (European Lipodystrophy Association: https://www.aelip.fr/). In patients with diabetes: the existence of Young Diabetics Association (https://www.ajd-diabete.fr/) and the Federation of Diabetics (https://www.federationdesdiabetiques.org/).

These associations contribute to better overall management of the disease by promoting cooperation between patients and caregivers and information for families (Appendix 2).The institutional websites www.orpha.net (ORPHA2348) and  https://endocrino-sat.aphp.fr/prisis/ also provide useful information for caregivers and families in France.

## Follow-up

### Objectives

- Establish, through a personalized follow-up, conditions that will foster the patient’s adherence to therapeutic measures and management of the disease and will best preserve the patient’s quality of life.
- Continue the multidisciplinary coordination of care and adapt to the evolution of the disease.
- Adapt treatments based on their effectiveness and the patient’s tolerance.
- Prevent complications of the disease.

### Professionals involved (and coordination arrangements)

- Follow-up is multidisciplinary and coordinated between the general practitioner, specialist physicians and professionals from the paramedical and social fields, in a manner adapted to the evolution of the disease (see “Professionals involved (and coordination)” section).
- The modalities and frequency of joint follow-up between professionals depend on the severity of the disease.

### Frequency and content of consultations

Follow-up of patients with Dunnigan syndrome should be carried out in cooperation with a reference center/competence center.

The frequency of consultations must be adapted to each patient’s situation: at least every six months in pediatrics and annually in adults, in coordination with the patient’s general practitioner. It will be more frequent in case of metabolic and/or cardiovascular complications.

If metreleptin treatment is initiated, a consultation should generally be scheduled after one month. Follow-up will then be scheduled quarterly for the first year in order to adapt the treatment according to the associated metabolic complications and to continue the patient’s therapeutic education.

In case of pregnancy, follow-up will be monthly, in parallel with obstetrical follow-up.

During each consultation, the following clinical elements will be examined:

- Weight, height, BMI, blood pressure, smoking status.
- Appetite (hunger, satiety), diet and physical activity.
- Pubertal stage.
- Compliance with treatments and checking for possible side effects.
- In case of diabetes, a discussion on self-monitoring of capillary glucose levels, checking for micro and macrovascular complications associated with diabetes, checking for lipodystrophy at insulin or metreleptin injection sites.
- In case of liver damage, checking for clinical signs of hepatocellular failure, portal hypertension and hepatomegaly.
- Checking for clinical signs of hypertriglyceridemia flare-ups (eruptive xanthomatosis, abdominal pain suggestive of pancreatitis).
- Checking for signs of cardiovascular disease: dyspnea (NYHA stage), chest pain, palpitations, syncopal malaise, signs of heart failure, signs of peripheral vascular disease.
- Checking for myalgia, arthralgia, clinical arguments for peripheral neuropathy or muscular dystrophy, static disorder.
- In adolescents and women, checking for irregular menstrual cycles and signs of hyperandrogenism. Contraception, fertility and pregnancy issues are to be addressed.
- Checking for signs of psychological suffering of the patient and within his/her close circle.
  The following will be scheduled for patients if needed:
- Specialized consultation (diabetology, cardiology, hepatology, gynecology, neurology, rheumatology, other) and management adapted to the clinical symptoms.
- Consultation with a dietician (dietary difficulties, overweight, carbohydrate intolerance, diabetes, hypertriglyceridemia).
- Consultation with a psychologist: psychological support
- Consultation with a social worker.

### Additional investigations

The following biological tests should be carried out systematically at least once a year and adapted to the progress of the disease and associated complications:

- HbA1c, fasting blood glucose
- Hemogram-platelets
- Blood ionogram, creatinine
- AST, ALT, PAL, GGT, total bilirubin
- Total cholesterol, triglycerides, HDL-cholesterol, LDL-cholesterol
- CPK

Microalbuminuria/creatininuria from urine sample (or 24-h proteinuria depending on the severity of renal damage)

- Leptinemia according to the clinical situation
- Insulinemia or C-peptide in case of insulin therapy (indication/follow-up of metreleptin treatment)

In case of diabetes, in addition to microalbuminuria/creatininuria, the following tests should be performed according to current recommendations:

- HbA1c every 3 months
- A fundus examination or fundus pictures at least once a year (to be adapted according to the stage of retinopathy)
- Screening for macrovascular complications

In case of hepatomegaly or liver biology disturbances and at the time of diagnosis, a liver morphological examination should be carried out (at least a liver ultrasound, ideally a metabolic MRI). Liver fibrosis should be evaluated using a Fib4 score and/or elastometry. Abnormal tests should be checked every six months for biology and annually for morphology in the absence of cirrhosis. The advice of a specialist in hepatology should be sought if necessary (see “Hepatic complications” section).

A cardiovascular check-up should be done at least once a year; adapted to clinical examination and the stage of the disease (see Appendices 4 and 5). It should include at least a resting electrocardiogram and trans-thoracic echocardiogram upon discovery of the disease. An early (starting at age 30) myocardial ischemia test and/or coronary scan should be proposed if there is a family history of atherosclerosis and/or other cardiovascular risk factors.

It may be necessary to look for sleep apnea syndrome depending on the warning signs, particularly asthenia.

An electromyogram is prescribed if there are clinical arguments for neuromuscular involvement.

Other examinations to assess the percentage of fat mass, its distribution, and muscle damage may be useful (DEXA, impedancemetry, full-body MRI). These specialized examinations are usually carried out in a reference or competence center.

The prescription of osteo-articular imaging examinations is based on clinical rheumatological symptomatology.

In case of menstrual cycle irregularities, an evaluation of the gonadotropic axis (LH, FSH and estradiol) will be proposed. In case of hyperandrogenism, total testosterone, SHBG and AMH should be measured at the beginning of the cycle. Depending on the results of this initial assessment, additional examinations are scheduled (pelvic ultrasound, pituitary MRI, anterior pituitary axes, etc.).

For Dunnigan syndrome and pregnancy, see Section Gynecological care. In men, in case of erectile dysfunction and/or decreased libido, an evaluation of the gonadotropic axis (LH, FSH, total testosterone and SHBG) is proposed.

## Data Availability

Not applicable.

## Appendix 1: Female morphotype associated with Dunnigan syndrome

Typical morphotype is associated with atrophy of subcutaneous adipose tissue of the limbs and trunk (lipoatrophy), with accumulation of adipose tissue in the face, neck, intra-abdominal region, as well as in axillary and pubic regions. Clinical presentation may suggest Cushing’s syndrome. The subcutaneous lipoatrophy reveals apparent veins and accentuates muscle mass (e.g. hypertrophic calves). The general morphotype is pseudo-athletic with broad shoulders.

## Appendix 2: Contacts

► PRISIS Network – French Competence and Reference Centers

**Table:**

http://endocrino-sat.aphp.fr/prisis
Dr Baron S., Nantes, France
Dr Bellanné-Chantelot C., Paris, France
Prof. Beltrand J., APHP, Paris, France
Prof. Brue T., Marseille, France
Prof. Carel JC., Paris, France
Prof. Cavé H., Paris, France
Prof. Delemer B., Reims, France
Dr Dubois-Laforgue, D., Paris, France
Prof. Gautier JF., Paris, France
Prof. Germain N., Saint-Etienne, France
Prof. Gourdy P., Toulouse, France
Prof. Hartemann A., Paris, France
Dr Jellimann S., Nancy, France
Dr Jéru I., Paris, France
Prof. Kerlan V., Brest, France
Prof. Kessler L., Strasbourg, France
Prof. Nicolino M., Lyon, France
Prof. Nobécourt E., La Réunion island, France
Dr Nivot-Adamiak S., Rennes, France
Dr Prevost G., Rouen, France
Prof. Rigalleau V., Bordeaux, France
Prof. Rodien P., Angers, France
Prof. Tauveron I., Clermont-Ferrand, France
Prof. Vantyghem MC., Lille, France
Prof. Vigouroux C., Paris, France

► Patient Associations

**Table:**

**French Lipodystrophy Association (AFLIP;** ***Association Française des Lipodystrophies*****)**
14 Rampe des Ginestes 04,860 Pierrevert
Website: https://fr-fr.facebook.com/pg/AFLIP02/
Website: https://www.alliance-maladies-rares.org/
**Young Diabetics Association (AJD;** ***Aide aux Jeunes Diabétiques*****)**
https://www.ajd-diabete.fr/
contact: ajd@ajd-educ.org
**French Federation of Diabetics (FFD; *****Fédération Française des Diabétiques*****)**
https://www.federationdesdiabetiques.org/
contact: federation@federationdesdiabetiques.org

►General information:

**Table:**

Portal for rare diseases and orphan drugs: http://www.orpha.net
French Rare Endocrine Diseases Network: http://www.firendo.fr/
French National Health Authority: https://www.has-sante.fr/
French Endocrinology Society: http://www.sfendocrino.org/
French Society of Pediatric Endocrinology and Diabetes: https://www.sfedp.org/
Francophone Diabetes Society: https://www.sfdiabete.org

## Appendix 3: Initial workup for the diagnosis of Dunnigan syndrome

**Table:**

*Clinical examination:*
Peripubertal onset of partial lipodystrophy
Measurement of skin folds (if possible with Harpenden caliper)
Measurement of body mass index and waist circumference
Growth curve in childhood, staturo-pubertal assessment
Cardiovascular examination
Neuromuscular examination
Checking for polycystic ovary syndrome in women (hirsutism, menstrual disorders)
Checking for hepatomegaly, possible signs of cirrhosis
Checking for sleep apnea syndrome
Creation of a family tree
*Genetic:*
Search for a *LMNA* gene pathogen variant, using a NGS Lipodystrophy panel
Screening of relatives
*Biological:*
Fasting blood glucose
Fasting insulin (C-Peptide in case of insulin therapy)
Blood glucose and insulin levels under 75 g OGTT in the absence of diabetes
Anti-GAD, anti-IA2, anti-insulin autoantibodies if diabetes
LDL-cholesterol, HDL-cholesterol, triglycerides
Lipase if elevated triglycerides > 5 g/L and/or abdominal pain
AST, ALT, GGT, PAL
Total bilirubin, albumin, platelets, hemostasis functions
Measurement of the Fib4 score: [Age (years) x AST (IU/L)/Platelets (10^9^/L) x √ < [ALT (IU/L)]
Leptin
CPK
*Hormonology in women:*
FSH, LH, E2, Testosterone, AMH, SHBG
*Morphological examinations:*
DEXA: distribution and % of body fat
Electrocardiogram
Blood pressure measurement
Screening for coronary artery disease and/or cardiomyopathy (Appendix 5)
Hepatic ultrasound
Elastometry if hepatomegaly, or ultrasound abnormalities
*Specialized consultations as needed:*
Dietary consultation
Cardiology consultation
Hepatology consultation
Gynecology consultation
Neuro-myology consultation
Psychology consultation

The initial workup can be modulated and other tests can be discussed based on the clinical context.

## Appendix 4: Patient follow-up after diagnosis in Dunnigan syndrome

**Table:**

*Multidisciplinary and personalized follow-up in coordination with a reference or competence center*
Frequency of consultations has to be adapted to the results of the initial workup (see Appendix 3) and to each patient’s situation, at least once a year in adults and every 6 months in children in the absence of complication
***Clinical examination:***
Measurement of weight, height, body mass index, pubertal stage in children/adolescents, blood pressure
Appetite, diet and physical activity, smoking status
Compliance for treatments, potential side effects
Checking for:
signs of hypertriglyceridemia flare-ups (eruptive xanthomatosis, abdominal pain suggestive of pancreatitis)
signs of cardiovascular disease: dyspnea (NYHA staging), chest pain, palpitations, syncopal malaise, signs of heart failure, of peripheral vascular disease, of sleep apnea syndrome
myalgia, arthralgia, signs of peripheral neuropathy, muscular dystrophy or static disorder
signs of psychological suffering from the patient and his/her relatives
*In adolescents girls and women:*
Checking for spaniomenorrhea and hirsutism
Contraception, fertility and pregnancy issues are to be addressed
*In case of diabetes:* Checking for self-monitoring of glucose levels, for micro and macrovascular complications
*In case of liver damage:* Checking for hepatomegaly, signs of hepatic failure or portal hypertension
***Biological tests/Paraclinical investigations:***
Frequency to be adapted to the disease progression and its associated complications (at least once a year):
HbA1c, fasting blood glucose, hemogram-platelets, blood ionogram, creatinine
AST, ALT, PAL, GGT, total bilirubin, creatine kinase
Total cholesterol, triglycerides, HDL-cholesterol, LDL-cholesterol
Microalbuminuria/creatininuria from urine sample (or proteinuria depending on the severity of renal damage)
Leptinemia, fasting insulin (C-Peptide if insulin therapy) if needed (indication or follow-up of metreleptin treatment)
*In case of diabetes*, according to current guidelines:
HbA1c every 3 months
Fundus examination or pictures at least once a year (to be adapted according to the stage of the retinopathy)
Screening for macrovascular complications
*In case of hepatomegaly or liver biology disturbances at diagnosis:*
Liver ultrasound, metabolic MRI if available, elastometry, Fib4 score
*Cardiovascular check-up* adapted to the stage of the disease (see Appendix 5)
*Regarding pregnancy*, see “Gynecological care” section
***Other investigations**** if needed, based on symptomatology*
Electromyogram, osteo-articular imaging examinations
DEXA, impedancemetry, full-body MRI
LH, FSH, estradiol, total testosterone, SHBG and AMH at the beginning of the cycle if menstrual cycle irregularities or hirsutism in women, or in case of erectile dysfunction in men, with imaging investigations if needed
***Specialized consultations**** if needed, including*: diabetology, cardiology, hepatology, gynecology, neurology, rheumatology, consultation with a dietician, a psychologist, a social worker

The follow-up can be modulated and other tests can be discussed based on the clinical context.

## Appendix 6: Modalities of treatment with metreleptin in France

### Prescribing and administration modalities

- A booklet for health professionals is available on the ANSM website (ansm.sante.fr). It specifies the prescription, safety and dispensing information for the product.
- It is strongly recommended that the prescription is validated during a multi-disciplinary consultation meeting of the PRISIS Reference Center. This should be done in conjunction with the hospital pharmacist who will organize the dispensing of the medication.
- The doctor should comment and give the patient an information booklet, instructions for use and an information card mentioning the prescribed dose, all of which are available on the ANSM website. He should train the patient in the preparation and self-administration of the product, and during the treatment monitoring.
- The medication consists of a powder that needs to be kept cold and reconstituted for each use in water to make an injectable solution.
- The injection should be given daily, at the same time and without interruption
- The initial dose (for example, 5 mg or 1 ml/d in women weighing more than 40 kg) is adapted according to metabolic efficiency (HbA1c, triglycerides, liver disturbances) and tolerance (weight) after one month of treatment.

### Undesirable effects

Undesirable side-effects affect about 30% of patients: most commonly hypoglycemia in insulin-treated patients, weight loss and injection site reactions (erythema, urticaria).

Anti-metreleptin autoantibodies are frequently detected, but they are very rarely neutralizing. They should be measured if there is a significant decrease in the treatment efficacy.

Rare cases of lymphoma have been reported in patients with autoimmune generalized lipodystrophy, pancreatitis has been described (in patients with a history of acute pancreatitis, especially after discontinuation of metreleptin), as well as worsening of renal or liver function.

### Metreleptin is not recommended during pregnancy

The prescription of metreleptin should be re-evaluated every 6 months based on the efficacy of the treatment and the patient’s tolerance and compliance to it. It is strongly recommended that the continuation of treatment be re-evaluated at least every year during a multidisciplinary consultation meeting of the PRISIS Reference Center.

## Abbreviations

ACMG: American College of Medical Genetics and Genomics
AELIP: Association of Families Affected by Lipodystrophies in Spain, Europe and Latin America
AFLIP: French Lipodystrophy Association (Association Française des Lipodystrophies)
AJD: *Aide aux Jeunes Diabétiques* Young Diabetics Association
ALD: *Affection de Longue Durée* Long-term condition justifying 100% coverage by the French social security (main list of 30 diseases)
ALD31: *Affection de Longue Durée 31* Long-term condition justifying 100% coverage by the French social security (not on the list of 30 diseases)
ALP: Alkaline phosphatase
ALT: Alanine aminotransferase
AMH: Anti-Müllerian Hormone
ANSM: *Agence nationale de sécurité du médicament et des produits de santé* French National Agency for Medicine Safety
AP-HP: *Assistance Publique-Hôpitaux de Paris* Public Assistance—Paris Hospitals
APRI: Aspartate-amino-transferase to platelet ratio index
AST: Aspartate aminotransferase
ATU: Authorization for Temporary Use
BMI: Body Mass Index
CBC: Complete Blood Count
CPK: Creatine Phosphokinase
CT scan: Tomodensitometry
DEXA: Dual-energy X-ray absorptiometry
DPP4: Dipeptidyl Peptidase-4
ECG: Electrocardiogram
CPDPN: *Centre pluridisciplinaire de diagnostic prénatal*, Multidisciplinary Center for Prenatal Diagnostics
FDA: Food and Drug Administration
FFD: French Federation of Diabetics
Fib4: Fibrosis score 4
FPLD: Familial Partial Lipodystrophy
FPLD2: Familial Partial Lipodystrophy type 2
FSH: Follicle Stimulating Hormone
GGT: Gamma Glutamyl-Transpeptidase
GLP-1: Glucagon-Like Peptide-1
GOT: Glutamate Oxaloacetate Transaminase
GPT: Glutamate Pyruvate Transaminase
HAS: *Haute Autorité de Santé* French National Health Authority
HbA1c: Hemoglobin A1C
HBP: High Blood Pressure
HDL: High Density Lipoprotein
ICD: Implantable Cardioverter Defibrillator
IGF1: Insulin-like Growth Factor-1
LDL: Low Density Lipoprotein
LH: Luteinizing Hormone
*LMNA*: Lamin A/C gene
MDPH: *Maison Départementale des Personnes Handicapées* Regional Centers for Disabled Workers
MRI: Magnetic Resonance Imaging
NASH: Non-alcoholic steatohepatitis
NT pro-BNP: N-Terminal Pro-Brain Natriuretic Peptide
NYHA: New York Heart Association
OGTT: Oral Glucose Tolerance Test
OMIM: Online Mendelian Inheritance in Man
PCOS: Polycystic ovary syndrome
PAI: *Projet d’Accueil Individualisé* Individualized Welcome Project
PNDS: *Protocole National de Diagnostic et de Soins* French National Diagnostic and Care Protocol
PNNS: French National Nutrition and Health Program
PPS: *Projet Personnalisé de Scolarisation* Personalized Schooling Project
PRISIS: *Centre de Référence National des Pathologies Rares de l’insulino-sécrétion et insulin-sensibilité*, National Reference Center for Rare Diseases of Insulin Secretion and Insulin Sensitivity
RCP: *Réunion de Concertation Pluridisciplinaire* Multidisciplinary Coordination Meeting
SGLT2: Sodium-Glucose Cotransporter-2
SHBG: Sex-Hormone Binding Globulin

## Declarations

### Group statement

The National Diagnostic and Care Protocol (PNDS) for Dunnigan syndrome was developed in its first version by accredited reference and competence PRISIS centers, with methodological support from the French National Health Authority (HAS), in application of the provisions of the National Rare Diseases Plan 2005–2023. This new version is placed under the sole editorial responsibility of the PRISIS network.
